# American marten occupancy and activity patterns at the southern extent of their range in the eastern United States

**DOI:** 10.1002/ece3.10904

**Published:** 2024-02-05

**Authors:** Sarah Ashbrook, Paul Hapeman

**Affiliations:** ^1^ Department of Biology Central Connecticut State University New Britain Connecticut USA

**Keywords:** carnivore, distribution, marten, mesocarnivores, occupancy, Vermont

## Abstract

The relatively recent rediscovery of an American marten (*Martes americana*) population that was reintroduced over 30 years ago in southern Vermont provides an opportunity to investigate the relative importance of other mesocarnivores, and forest stand (e.g., DBH, downed logs, vertical structure) and habitat variables to their presence on the Green Mountain National Forest. Marten are state‐listed as an endangered species in Vermont and occur there at the southern extent of their range in the eastern United States. We collected detection data from camera surveys in 5 km^2^ units between 2019 and 2021 (December–April; *n* = 40 units, 238 cameras). We examined activity patterns and applied an occupancy modeling framework to the detection data to assess the relative importance of covariates at unit and camera levels and assess interactions of marten with other mesocarnivores. We did not find any unit‐level occupancy models with significant covariates that were better supported than the base model in the single‐season unit‐level analysis. Distance to the nearest release site was the covariate most supported for detectability at both spatial scales, and marten occupancy at the camera level was positively influenced by the amount of canopy cover. Two species interaction models did not indicate any positive or negative association beyond random with other mesocarnivores and activity patterns among mesocarnivores had substantial overlap. Marten recovery since the time of the reintroduction appears slow, and even 30 years later, the marten distribution is limited and suggests that dispersal is restricted at some level. We recommend a further investigation of the possible impact of other mesocarnivores to juvenile survival or other vital demographic rate (e.g., recruitment) in marten that were not explicitly measured in this study.

## Graphical abstract

Models of occupancy using detection data from camera surveys for American marten in southern Vermont indicated that detectability decreased with increasing distance from historical reintroduction sites. Unit‐level occupancy models containing covariates did not perform better than the base model that assumes occupancy is homogeneous among units. Marten appears to be restricted by some attribute (e.g. vital demographic rate) that was not measured in the study.

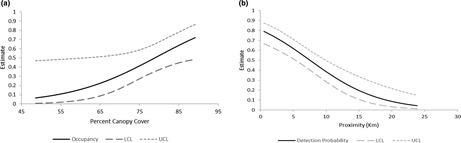

## 1 INTRODUCTION

Multiple drivers such as habitat type (Gompper et al., 2016), human disturbance (Kalle et al., 2013), climate (Zielinski et al., 2017), and species interactions (Guo et al., 2017), govern the occurrence of carnivores in complex ways that vary in importance between and within species and operate at different spatial scales (Mitchell & Hebblewhite, 2012). Further, occurrence is closely associated with specific life history activities such as breeding and foraging (Dechner et al., 2018; Uboni et al., 2017) or can reflect resource availability (Grassman Jr. et al., 2005).

Range boundaries often have markedly different conditions compared to interior parts of a species range and present notable challenges for resident carnivores. Their body sizes can be smaller at range boundaries compared to interior portions of their range and fundamental niches can be limited on at least one niche axis (Meiri et al., 2009). Carnivores that occur at range boundaries are less abundant (Brown et al., 1995) and have difficulty adapting to changing conditions (Brown, 1984; Whittington et al., 2022).

Biotic interactions such as competition and predation are predicted to be more important at range boundaries, particularly at low latitudes, and can constrain occurrence patterns (Aragón & Sánchez‐Fernández, 2013; Louthan et al., 2015; Sirén & Morelli, 2020). Competitive generalist predators (e.g., coyotes; *Canis latrans*) at range boundaries can potentially limit prey species to areas of reduced predation (Mott, 2010). Generalist predators also behave opportunistically by altering their diet and behavior in response to changes in the availability of different prey types, which may feedback on other subordinate predators and force them to alter their realized niche spaces (Glasser, 1982; Smith et al., 2018). Competition for food and suitable habitat by a competitively superior species may force a subordinate species into suboptimal habitat (Hairston et al., 1987; Jaeger, 1971a, 1971b, 1972; Kyaw et al., 2021; Vanek et al., 2013). Coexistence among carnivores in these areas is potentially possible through mechanisms that minimize intraguild competition and predation including spatial avoidance, minimizing dietary overlap, and temporal shifts in daily or seasonal activity patterns in the search for and acquisition of resources (Grassel et al., 2015; St. Pierre et al., 2006).

American marten (*Martes americana*) are small, elusive carnivores that are broadly distributed throughout the boreal forest zones of North America (Powell et al., 2003). They were considered common and widely distributed prior to the 20th century (Moruzzi et al., 2003) and their historical range in the eastern United States once extended as far south as northern Pennsylvania (Rhoads, 1903). Like other native furbearers including fisher (*Pekania pennanti*) and mink (*Neovison vison*), their numbers and distribution declined significantly in the northeastern United States (hereafter, the Northeast) from overtrapping and habitat loss (Brander & Books, 1973; Silver, 1957) and by the early 1900s, they were extirpated from most areas of the Northeast (e.g. Vermont, New Hampshire) except the Adirondacks of New York and northern portions of Maine (Krohn, 2012).

**FIGURE 1:**
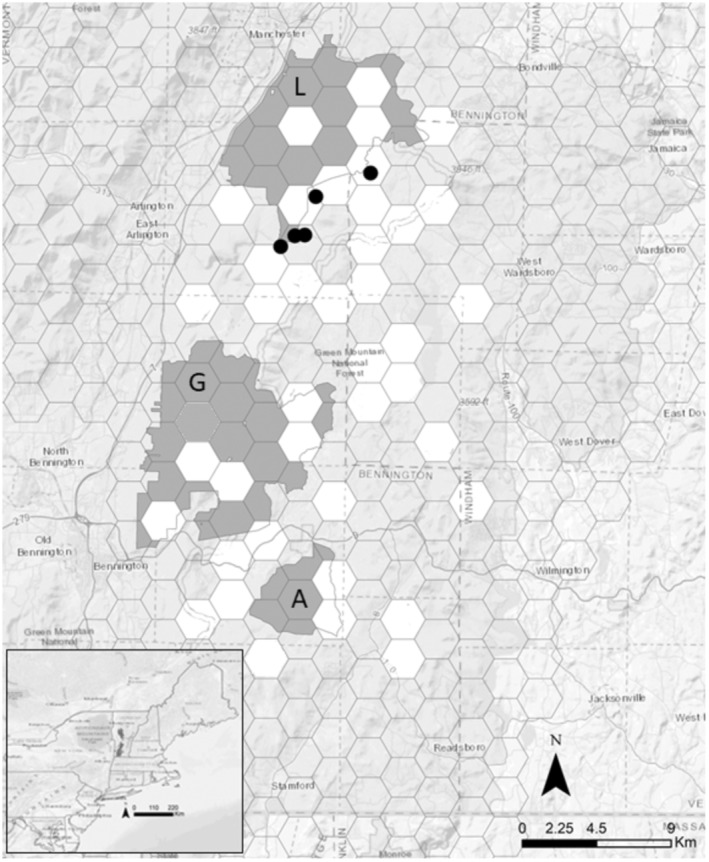
Map showing locations of units where camera surveys occurred between 2019 and 2021 (white). Black circles indicate sites where marten were released during the reintroduction between 1989 and 1991. The study area for camera surveys in Vermont is circled on the inset map. Landmarks include Lye Brook Wilderness (L), Glastenbury Wilderness (G), and Aiken Wilderness (A).

Vermont closed the marten trapping season and eventually listed the species as endangered in 1972 (10 V.S.A. Chap. 123‐2012). Active recovery of marten in Vermont included the reintroduction of animals from Maine (*n* = 104) and New York (*n* = 11) between October 1989 and December 1991 at five sites in southern Vermont (Distefano et al., 1990; Moruzzi et al., 2003; Royar, 1992; Figure 1). Despite considerable monitoring effort of the reintroduced marten by Vermont Fish and Wildlife (Royar, 1992), camera surveys from 1994–1995 and 1997–1998 resulted in only a few marten detections, and the reintroduction effort was later deemed unsuccessful (Moruzzi et al., 2003). One plausible explanation proposed for the assumed failure of the reintroduction was competition and predation by fishers (Moruzzi et al., 2003). Winter camera surveys conducted between 2015 and 2017 revealed a population of marten in the southern part of the Green Mountain National Forest (O'Brien et al., 2018) that may be a remnant of the original reintroductions (Aylward et al., 2019). This population currently represents the southernmost extent of the marten distribution on the east coast. As a state‐listed endangered species (10 V.S.A. Chap. 123‐2012) in Vermont, marten residing there require a thorough understanding of their distribution, habitat requirements, and limiting factors to effectively manage them.

Preferred habitat characteristics of marten are variable and largely dependent on age, sex, season, and population density (Buskirk & Powell, 1994; Clark et al., 1987). Characteristics associated with home range and habitat use by marten vary at different spatial scales that are relevant for their conservation (Chapin et al., 1998; Smith & Schaefer, 2002). Across the eastern part of their distribution, marten demonstrate an avoidance of open areas (Cheveau et al., 2013; Gosse et al., 2005) and landscapes lacking structural complexity as a method of minimizing the risk of predation (Eriksson et al., 2019). Marten prefers vertical and horizontal structural complexity that may be beneficial for hunting and provide protection from predators (Evans & Mortelliti, 2022). They have an affinity for a higher elevation when available (Sirén et al., 2016) including mature forest, canopy closure, mixedwood and softwood, and coarse woody debris (Godbout & Ouellet, 2010; Jensen, 2012; Lambert et al., 2017; Payer & Harrison, 2004).

Most studies that examined habitat features associated with marten in eastern North America have occurred in either interior parts of their range or in populations at the northern extent of their range. Little is known about the importance of co‐occurring mesocarnivores that interact with marten as competitors and predators at the southern extent of their range such as red fox (*Vulpes*), coyote, bobcat (*Lynx rufus*), and fisher (Bull & Heater, 2001; Pauli et al., 2022; Raine, 1981; Suffice et al., 2023; Vernam, 1987). Krohn et al. (1995, 1997) suggested that high fisher populations limit marten populations at the southern extent of their range. However, recent work by Manlick et al. (2017), Croose et al. (2019) suggests a large amount of spatial and temporal overlap between marten and fisher is possible.

In this study, we evaluated the relative importance of other mesocarnivores, forest stand variables, and habitat variables to occupancy and detectability (MacKenzie et al., 2005, 2006; MacKenzie & Royle, 2005) of marten at multiple spatial scales (Gompper et al., 2016; Lesmeister et al., 2015; Long et al., 2010). We focused primarily on marten habitat use at the 5 km^2^ unit level which coincided with the scale of the average home range size of male American marten home range and the development of a long‐term monitoring program. The specific objectives were to (1) provide camera and unit level estimates of occupancy and detectability for marten, (2) identify the relative importance of other mesocarnivores, forest stand variables, and habitat variables to marten occurrence in the study area, (3) identify relationships between marten occurrence and the presence of other mesocarnivores in the study area, and (4) compare activity pattern overlap of marten with other mesocarnivores detected in the study. We predicted that occupancy models for marten that included covariates associated with other mesocarnivore occupancy or detections would have greater support than models that included forest stand variables and other habitat variables measured in this study (Louthan et al., 2015). We also predicted that marten occupancy and detectability would be lower further away from reported release sites given the limited number of anecdotal reports of marten in southern Vermont. We predicted that occupancy and detectability would be negatively associated with the presence of coyote, red fox, gray fox (*Urocyon cinereoargenteus*), and bobcat (Martin et al., 2022) but not fisher (Manlick et al., 2017). We also predicted that marten would show substantial overlap in daily activity patterns with fisher based on the recent work of Manlick et al. (2017) but not with other mesocarnivores in our study.

## 2 STUDY AREA

The study area was located within the southern extent of the Green Mountain National Forest (GMNF) in southern Vermont and northwestern Massachusetts in the United States (Figure 1). The Green Mountain National Forest encompassed more than 1620 km^2^ in southwestern and central Vermont, forming the largest contiguous block of public land in the state (United States Department of Agriculture Forest Service (USFS), 2006). The study area was mountainous with a maximum elevation of 1220 m and included 3 federally designated wilderness areas (Lye Brook, George D. Aiken, and Glastenbury). The forest was composed primarily of northern hardwoods (~80%) including red oak (*Quercus rubra*), American beech (*Fagus grandifolia*), yellow birch (*Betula alleghaniensis*), red maple (*Acer rubrum*), and sugar maple (*Acer saccharum*). Coniferous stands were predominantly balsam fir (*Abies balsamifera*) and spruce (*Picea* spp.) and occur near wetland areas. Understory vegetation was highly variable and includes hobblebush (*Viburnum alnifolium*) and common witchhazel (*Hamamelis virginiana*; Siccama, 2014).

## 3 METHODS

### 3.1 Data collection

We collected detection data from camera surveys in 40 (*n* = 238 cameras), 5 km^2^ sample units over three winter field seasons (December–April) between 2019 and 2021. We surveyed each unit only once during the time we surveyed (units sampled yearly *n* = 15, 19, 6). The primary focus of the surveys was to detect marten, so we scaled the units to represent an area equivalent to the average home range size of male American marten (5 km^2^; Jensen, 2012). Several species were also important to document because of their potential for predation on or competitive interactions with marten including coyote, fisher, bobcat, and red and gray fox. We selected units within the study area based on random sampling with the constraint that the overall sample had to include units where the probability of marten was non‐zero and occurrence ranged from high to low (Jensen, 2012).

Within each sample unit, we used simple random sampling to place six motion‐triggered cameras (Moultrie 990is, P150s; Moultrie Products, Birmingham, AL and Bushnell Trophy Cam Aggressors; Bushnell Outdoor Products, Overland, KS) at sites to survey for marten. We expected that the use of camera arrays within the sample units would increase the likelihood of detecting marten during the surveys (O'Connor et al., 2017). At camera each site, we mounted cameras on trees one and a half to two meters above ground surface and aimed them toward the base of a bait tree to maximize the field of view. On the bait tree, we placed a single can of sardines in water wired approximately 1 m above the ground, and emptied another can at the base of the tree. To each bait tree, we applied a mixture of skunk essence (Wildlife Control Services, Granby, CT) and petroleum jelly (<15 mL) and separately, a curiosity scent (“Marten Magic”; Minnesota Trapping Supplies, Inc., Pennock, MN) containing peppermint‐anise oil extracts (<1.5 mL).

Camera surveys in units lasted 15 days divided into three consecutive 5‐day periods (total = 3 survey periods, 15 days), and following each 5‐day survey period, we re‐scented, re‐baited, and exchanged the data cards. The data resulted in detection histories with a 0 or 1 for each of the three survey periods (e.g., 010) for each unit. To maintain the independence of our units, we avoided carrying out surveys in adjacent units at the same time. We set cameras to operate for 24 h per day and recorded date, time, barometric pressure, and temperature at the time of all detections. Cameras were set to minimum delay with a three‐shot photo burst. Images were stored in Colorado Parks and Wildlife (CPW) Photo Warehouse 4.3 and reviewed by a minimum of two individuals; that recorded the date, time of day, and frequency of detections for each species. We also recorded notable behaviors (e.g. vigilance antipredator behavior such as upright posture, and looking around), weather conditions, and circumstances observed in the photos or at the time of data collection in the field. We listed images as unknown species in the CPW database and did not include them in the analysis if we could not identify the species.

### 3.2 Covariate data

We collected data on 14 covariates that were potentially associated with marten presence and detectability (Table 1 and Table S2). Covariate data was collected within a 30 m^2^ buffer around camera sites and within the entire units by extracting covariate data from polygons in ArcMap (Table 1). We recognize that simple detection or non‐detection of a mesocarnivore during a survey does not account for imperfect detection, so we included site occupancy estimates of mesocarnivores generated in the program PRESENCE 2.1.3.47 (Hines, 2006) as covariates. Data for the five covariates related to forest stand metrics (Table 1) was collected in the field at each camera site during summer 2019 following the Common Stand Exam Field Guidelines (USFS, 2015).

**TABLE 1:** List of covariates used to model marten occupancy and detectability in MARK from camera survey data collected between 2019 and 2021 in the Green Mountain National Forest of Vermont.

Covariate	Level	Prediction	Description
(1) PerFor	U (*ψ*)	Positive	Percent (Per) of unit classified as forest^a^
(2) Habitat type e.g. PerDec, PerMix	U (*ψ*, *p*)	Positive for forest types	Percent (Per) of 30 m raster cells within units classified as a specific habitat type^a^ including Deciduous (PerDec), Mixed, Conifer, Human Disturbance, Wet Woody, Water
(3) PerCC	U (*ψ*, *p*)	Positive	Canopy cover (CC) percent derived from percent tree canopy cover layer, values range 0%–100%^a^
(4) Can_Clos	U, C (*ψ*)	Positive	Value of canopy closure between 0–100 using a spherical densiometer^b^
(5) Vert_Struc	U, C (*ψ*)	Positive	Classification of vertical distribution of tree size from Common Stand Exam Field Guide USFS (2015)^b^
(6) DBH	U, C (*ψ*)	Positive	Mean value (cm) for trees within a radius of camera with radius defined using BAF 20 prism^b^
(7) D_Logs	U, C (*ψ*)	Positive	Number of downed logs of decay classes 1–2 from Common Stand Exam Field Guide USFS (2015)^b^
(8) BAF	U, C (*ψ*)	Positive	Value in m^2^/ha using a Basal Area Factor (BAF) 20 prism^b^
(9) Dist	U, C (*ψ*, *p*)	Negative	Distance from each units centroid or camera location to the closest reported release site (km)^c^
(10) Elev	U (*ψ*)	Positive	The 2‐D mean elevation within each sampling unit^d^
(11) TRI	U (*ψ*)	Positive	Topographic Relief Index. The 3‐D slope of the landscape within each sampling unit. Index is derived using ArcGIS 10.8 (ESRI) to extract raster cell values from the USGS National Elevation Dataset (NED) 1/3 arc second Digital Elevation Model 10 m raster^d^
(12) Snow	U, C (*ψ*, *p*)	Positive	Unit locations overlaid on daily climate data maps from the National Weather Service to collect average historic snow depth values at each location for the duration of each survey period^f^
(13) Mesocarnivores			Fisher (Fish), coyotee (Coy), fox (fox), bobcat (Bob)
e.g. FishPres, CoyPres	U (*ψ*, *p*)	Negative except fisher	Whether a species was detected (e.g. Coy Pres) during any survey period (1 present, 0 absent)^g^
e.g. FishOcc, CoyOcc	U, C (*ψ*, *p*)	Negative except fisher	Site occupancy estimate from base model Psi(.), p(.) for other mesocarnivores at cameras and in units^g^. Site estimates produced using the program Presence 2.13.47 Hines (2006)
			Site estimates produced using the program Presence 2.13.47 Hines (2006)

*Note*: Level (U, Unit; C, Camera) indicates the scale at which the covariate data was included in the modeling process. The parameter for the covariate is either *ψ* (Psi) or *p* (Detectability). Positive or Negative, predicted relationship of covariate to occupancy or detectability (e.g., Positive implies that as covariate values increase so do values for occupancy or detectability).

Data sources: a—National Land Cover Database 30 m resolution, b—Field current study, c—Historical reintroduction map, d—USGS National Elevation Dataset, e—Vermont Center for Geographic Information, f—https://www.weather.gov/btv/climatemaps, g—Camera.

### 3.3 Occupancy analyses

We conducted the occupancy analyses at several different spatial scales. We initially performed a single‐season, single‐species occupancy analysis at the unit level with covariates. For this analysis, we pooled detection data from the six cameras within units to produce a single detection survey for the entire unit. We also performed a hierarchical analysis similar to Pavlacky Jr. et al. (2012) using the multiscale occupancy estimation (e.g., *ψ*(.), *θ*(.), *p*(.)) in MARK 10.1 (White & Burnham, 1999). This approach allowed us to simultaneously examine the impact of covariates on local occupancy (*θ*) and detectability (*p*) at the camera site level while holding unit‐level occupancy (*ψ*) constant. We assessed species interactions by including detections of other mesocarnivores during any survey period and site occupancy estimates for each mesocarnivore in the single season, single species models at the unit level. Finally, we used the two‐species conditional occupancy model of Richmond et al. (2010) to produce estimates of a Species Interaction Factor (SIF; Φ) where SIF = *ψ* ^A^ × *ψ* ^BA^/(*ψ* ^A^ × (*ψ* ^A^ × *ψ* ^BA^ + (1 − *ψ* ^A^) × *ψ* ^Ba^)), *ψ* ^A^ = occupancy probability for species A, *ψ* ^BA^ = occupancy probability for species B given species A is present, and *ψ* ^Ba^ = occupancy probability for species B given species A is absent. Species are considered to be acting independently when SIF = 1.

### 3.4 Modeling approach

Due to the considerable number of covariates (and resulting occupancy models) in the study, we adopted a sequential modeling approach in the single‐season and multiscale analyses using single covariates (standardized within MARK). For each analysis, we first modeled each focal parameter of interest (*ψ*, *θ*, or *p*) with covariates separately while other model parameters were held constant (e.g. *ψ*(Coyote), *p*(.); Figure S1). We then selected top models in this first stage using Akaike's Information Criterion adjusted for small sample size (AICc; Burnham & Anderson, 2002). We used a more relaxed inclusion threshold (ΔAICc < 5) to select the top models for each parameter of interest to prevent the removal of covariates that may be important in combination with other covariates in more complex models (Morin et al., 2020). Covariates from the top models selected in the first stage were then included in a second modeling step. In the second modeling step (Figure S1), we combined covariates in all possible combinations (Doherty et al., 2012) from top models in the first step to produce an overall set of models that included covariates on the combined parameters (e.g., occupancy and detectability; *ψ*(Coyote), *p*(PerCC)). We based our interpretations on the single season and multiscale analyses only from models in this overall set that were within ΔAICc < 5 of the top model.

We limited the complexity of the models to avoid overfitting by including no more than three covariates (in addition to intercepts) Donovan and Hines (2007) and did not include covariates in the same model that showed evidence of collinearity (*r* Pearson > .50; Wilson & Schmidt, 2015). We produced model‐averaged estimates for each parameter in MARK using all models in the overall set with a ΔAICc < 5. We established the relative importance of each covariate in the overall model sets by summing the Akaike weights (*w*) of the models (ΔAICc < 5) where each covariate had a significant effect. We considered the effect of covariates to be significant if the 95% confidence interval for their beta estimates did not include zero.

### 3.5 Activity patterns

We used a slightly longer period of time for independence (>1 h apart) than Kautz et al., 2021 (30 min) to assess activity patterns of mesocarnivores from camera surveys for each species of interest (Foresman & Pearson, 1999). The detection times from the camera data that were entered into the CPW database for each species were exported as radians and imported into the Overlap package in R (Ridout & Linkie, 2009) for analysis. We assessed overlap among mesocarnivores using the Coefficient of Overlap (Dhat_4_ estimator) from Ridout and Linkie (2009). Species that have activity patterns that completely overlap have a value of 1 and with no overlap, a value of 0. The 95% confidence intervals around the Dhat_4_ estimates were developed using 200 bootstrap replicates using the bootCI function in Overlap.

## 4 RESULTS

### 4.1 Unit level

We detected marten at 18 of 40 units (45%) during 3600 camera trap nights. The overall top model set for the single‐season analysis included five models with a ΔAICc < 5 (Table 2). Model averaged estimates of occupancy and detectability over the five models were *ψ* = 0.579 (SE = 0.113, 95% CI = 0.356–0.774) and *p* = .609 (SE = 0.109, 95% CI = 0.387–0.793). Beta estimates for model parameters are found in Table S1. The base model (.) that assumes occupancy does not vary among units carried the most weight on occupancy (0.640) when summed across models. Distance to release site had the greatest relative importance for detectability (0.608) when summing across models and had a significant beta estimate. Other covariates on detectability that had some support were the percentage of deciduous forest (+) and the percentage of coniferous forest (−) in the unit.

**TABLE 2:** Top‐ranked overall single season unit level models (ΔAICc < 5) generated in MARK for American marten from detection data collected between 2019 and 2021 on the Green Mountain National Forest of southern Vermont.

Model	AICc	ΔAICc	Weights	#par	−2log(L)
Psi(.), p(Dist+PerDec)	106.026	0	0.352	4	96.884
Psi(Dist), p(PerCC)	106.525	0.499	0.274	4	97.382
Psi(.), p(Dist+PerCC)	107.523	1.497	0.167	4	98.380
Psi(.), p(Dist+PerCon)	107.653	1.627	0.156	4	98.510
Psi(Dist), p(PerDec)	109.865	3.838	0.052	4	100.722

*Note*: Psi = occupancy, *p* = detectability, AICc and ΔAICc = values for Akaike Information Criterion adjusted for small samples and relative to top model. Weights = model weight, and −2Log(L) = log‐likelihood of model, #Par = number of parameters in model.

**FIGURE 2:**
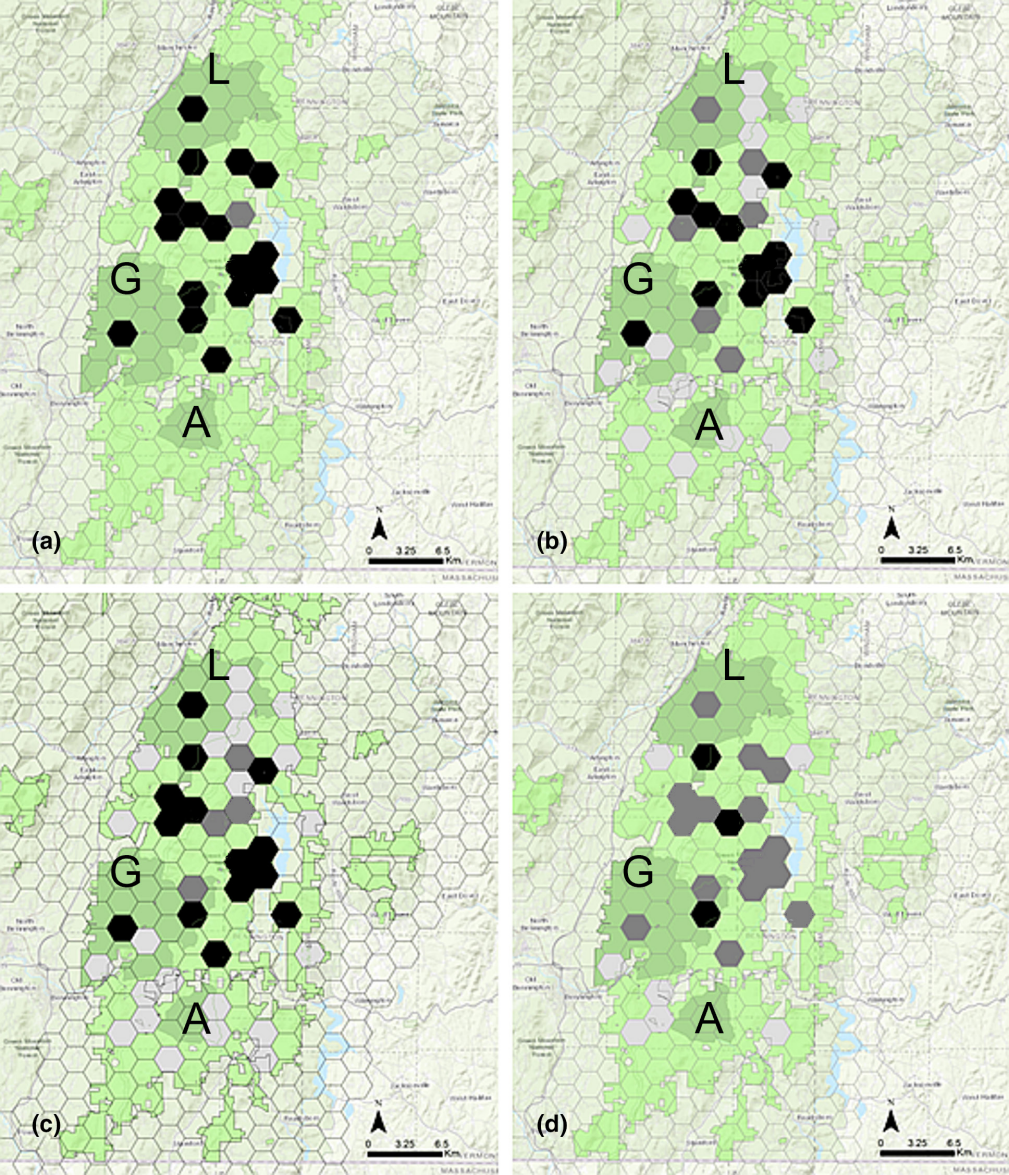
Sample units showing co‐occurrence of mesocarnivores in units on the Green Mountain National Forest of Vermont. Landmarks include Lye Brook Wilderness (L), Glastenbury Wilderness (G), and Aiken Wilderness (A) in darker green. Species include (a) marten and fisher (b) marten and fox (c) marten and coyote, and (d) marten and bobcat. Black = both species detected, dark gray = marten only, and light gray = fisher, fox, coyote, or bobcat only.

Mesocarnivores of interest were also detected in units during camera surveys including fisher (38/40 units), coyote (35/40 units), fox (28/40 units), and bobcat (10/40). Co‐occurrence in units where marten were detected was greatest for fisher (17/18 units; Figure 2a) followed by fox (12/18; Figure 2b) coyote (13/18; Figure 2c), and bobcat (3/18; Figure 2d). The species interaction factor estimates (Φ) at the unit level from the species interaction models ranged from 0.563 between marten and bobcat to 0.994 between marten and fisher (Table 4). Confidence intervals for all estimates of phi (Φ) encompassed one and did not indicate any significant interactions with occupancy between marten and other mesocarnivores at the unit level in the study (Table 4).

### 4.2 Camera level

**FIGURE 3:**
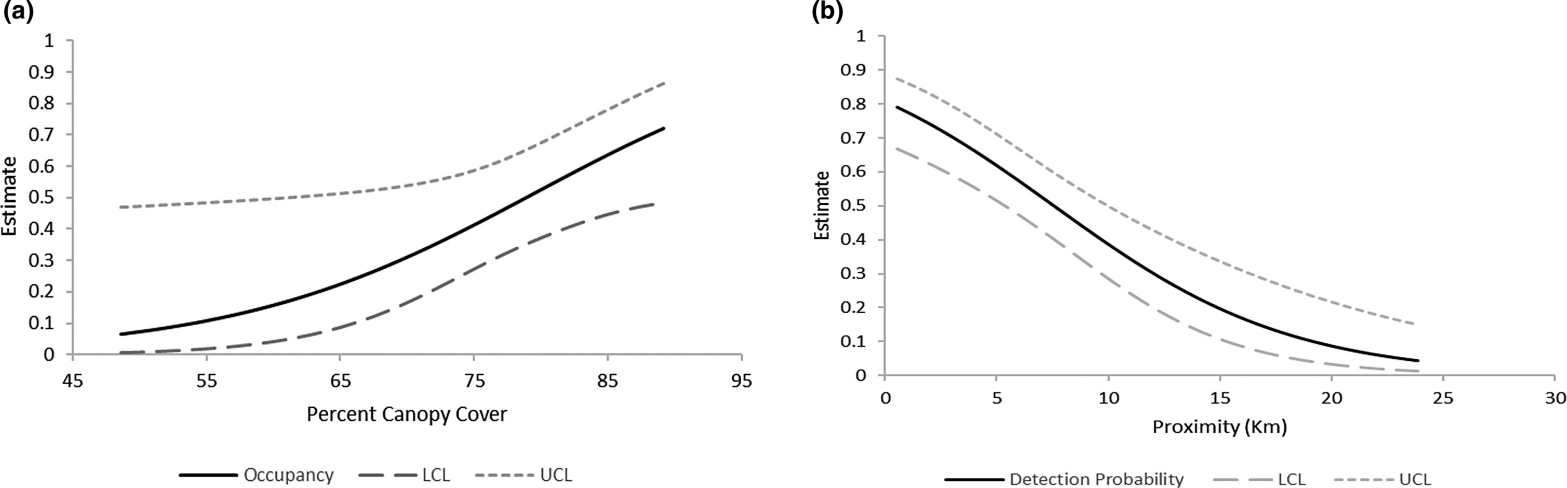
Relationships of covariates from multiscale occupancy analysis of marten (*Martes americana*) for Season 2 data collected on the Green Mountain National Forest between 2019 and 2021. (a) Change in local occupancy (*θ*) for different values of canopy cover (PerCC) and (b) Change in local detectability with Distance to nearest marten release site (Dist).

We detected marten at 93/238 cameras (39%). The analysis of local occupancy (*θ*) and detectability (*p*) from the multiscale models resulted in only a single model with a ΔAICc < 5 where covariates in the model were significant (Table 3; *ψ*(.), *θ*(PerCC), *p*(Dist)). Real parameter estimates from this model included *ψ* = 0.560 (SE = 0.099, 95% CI = 0.365–0.738), *θ* = 0.499 (SE = 0.066, 95% CI = 0.372–0.629), *p* = .389 (SE = 0.057, 95% CI = 0.283–0.507). Beta estimates for model parameters are found in Table S1. Percent canopy cover had a positive influence on local occupancy while camera level detections decreased the further cameras were located from documented release sites (Figure 3).

**TABLE 3:** Top‐ranked overall set of models (ΔAICc < 5) for multiscale analysis (*ψ*, *θ*, *p*) generated in MARK.

Model	AICc	ΔAICc	Weights	−2Log(L)	#par
psi(.), theta(PerCC), p(Dist)	378.589	0	0.568	1	5
psi(.), theta(Vert_Struc), p(Dist)	380.900	2.3115	0.179	0.315	5
psi(.), theta(CoyOcc), p(Dist)	381.109	2.5205	0.161	0.284	5
psi(.), theta(.), p(Dist)	382.221	3.6318	0.092	0.163	4

*Note*: Detection data from American marten collected between 2019 and 2021 in the Green Mountain National Forest of southern Vermont.

Psi = unit level occupancy, theta (*θ*) = local occupancy, *p* = camera level detectability, AICc and ΔAICc = values for Akaike Information Criterion adjusted for small samples and relative to top model. Weights = model weight, and −2Log(L) = log likelihood of model, #Par = number of parameters in model.

Species interaction factor estimates (Φ) at the camera site level from the species interaction models ranged from 0.968 between marten and fox to 2.125 between marten and bobcat (Table 4). Confidence intervals for all Φ estimates encompassed one and did not indicate any significant interactions with occupancy between marten and other mesocarnivores at the camera level in the study (Table 4).

**TABLE 4:** Species interaction factors (Φ; Richmond et al., 2010) at the unit and camera levels between marten and other mesocarnivore species of interest.

Species comparison	Level	Φ	SE	Lower	Upper
Marten‐Bobcat	U	0.563	0.431	−0.281	1.407
	C	2.125	0.949	0.265	3.984
Marten‐Fisher	U	0.993	0.042	0.910	1.078
	C	1.084	0.075	0.936	1.231
Marten‐Fox	U	0.988	0.137	0.719	1.257
	C	0.968	0.288	0.403	1.533
Marten‐Coyote	U	0.938	0.097	0.747	1.129
	C	1.125	0.156	0.819	1.431

*Note*: Estimates generated in MARK are from detection data collected between December and April during the years 2019 through 2021 in the Green Mountain National Forest of southern Vermont. Lower and Upper represent 95% confidence intervals around the Φ estimates. For each comparison, marten were assumed to be the subordinate predator. Level (U = Unit, C = Camera).

Abbreviation: SE, standard error.

### 4.3 Activity

**FIGURE 4:**
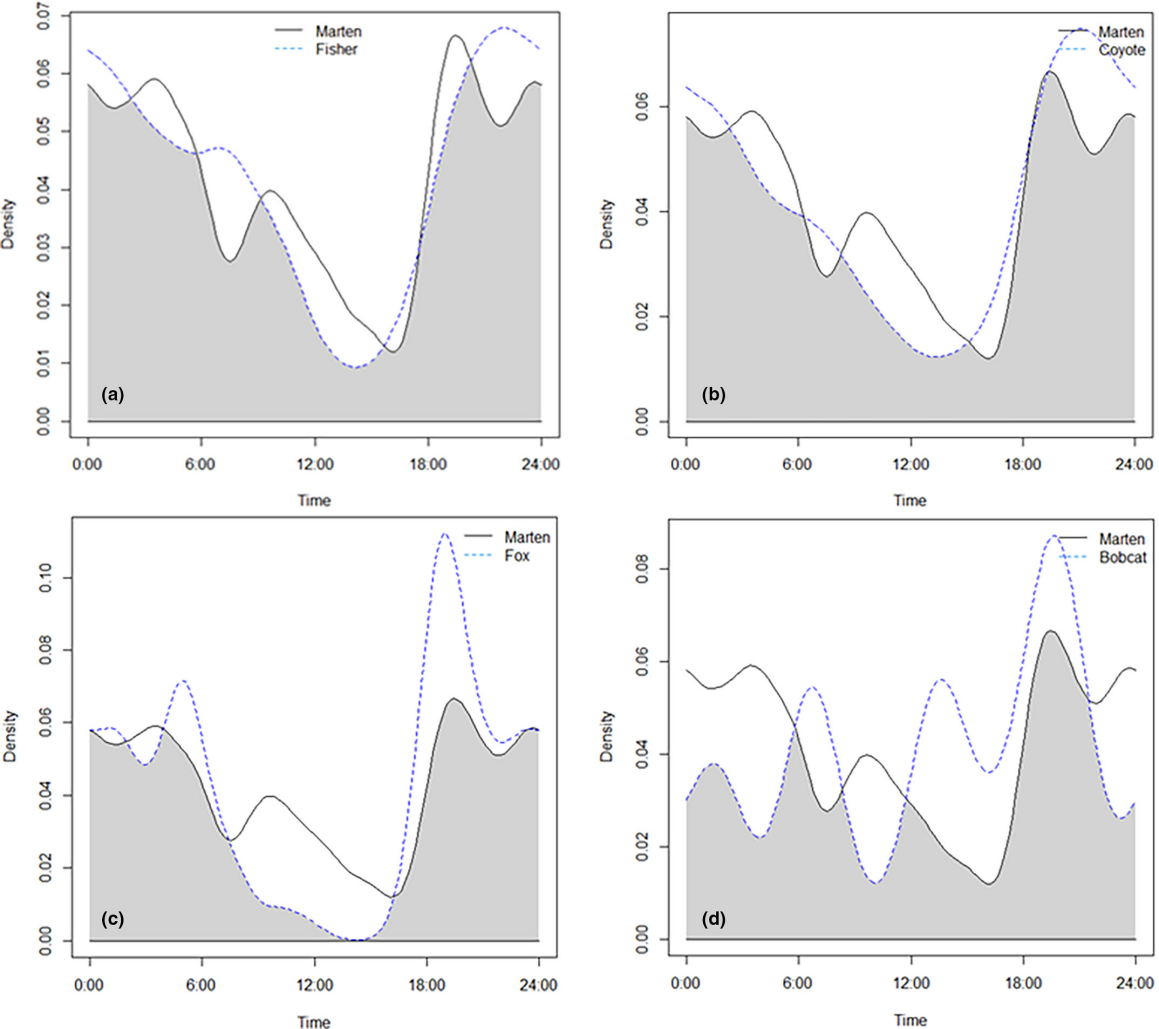
Activity patterns of marten showing overlap and peak time periods with other mesocarnivores including (a) fisher, (b) coyote, (c) fox, and (d) bobcat. Figures produced in the R package Overlap version 0.3.4 (Ridout & Linkie, 2009).

All mesocarnivores were predominantly nocturnal with peak activity occurring between early evening and midnight. Marten activity was high until approximately 04:00 (Figure 4). Overlap coefficients were generally high (Dhat_4_ = 0.701 to 0.929; Table 5). The largest overlap for marten with another species occurred with fisher (Dhat_4_ = 0.897; Table 5 and Figure 4).

**TABLE 5:** Estimates of activity pattern overlap using the Coefficient of Overlap (Dhat_4_) from Ridout and Linkie (2009).

Comparison	Dhat_4_	Lower CI	Upper CI
Marten‐Fisher	0.897	0.847	0.96
Marten‐Coyote	0.891	0.849	1.00
Marten‐Fox	0.801	0.737	0.894
Marten‐Bobcat	0.715	0.565	0.883
Fisher‐Coyote	0.929	0.907	1.00
Fisher‐Fox	0.794	0.715	0.852
Fisher‐Bobcat	0.705	0.554	0.895
Coyote‐Fox	0.794	0.765	0.902
Coyote‐Bobcat	0.735	0.605	0.925
Fox‐Bobcat	0.701	0.576	0.899

*Note*: Lower and upper CI = 95% confidence intervals on Dhat_4_ estimates. Detection data from cameras considered independent if photos were taken >1 h apart. Sample sizes for each species were marten (*n* = 160), fisher (*n* = 903), bobcat (*n* = 19), coyote (*n* = 123), and fox (*n* = 142).

## 5 DISCUSSION

We predicted that occupancy and detectability of marten at the southern edge of their distribution in Vermont would have a negative association with other mesocarnivores and those associations would be more important compared to forest stand variables and habitat variables measured in the study. We found little evidence to support these predictions; the majority of the top overall models in the various analyses did not contain covariates related to other mesocarnivore occupancy or detectability.

Distance to the nearest release site was the most important covariate to marten detectability at unit and camera levels. Given their reported dispersal distances (Johnson et al., 2009), we would have expected marten to have expanded further from the release sites in the GMNF in the 30 plus years since they were reintroduced. The survey results provide evidence that marten has been recovering in the central portion of the GMNF since the time of the reintroductions (1989–1991), but that recovery appears slow. Follow‐up monitoring efforts after the reintroduction revealed that most marten dispersed within 16 km (average 6.6 km) from the release sites (Royar, 1992). We did not detect marten beyond 12 km from the release sites, and marten occupancy and detectability decreased with increasing distance from the nearest release site. Marten was also not detected during camera surveys in units further south in our study area (O'Brien et al., 2018) or at several sites further north of the study area, including another release site used during the 1989–1991 reintroduction (P. Hapeman 2018, unpublished data). A similar delayed recovery has been observed for marten in Wisconsin and has been attributed to the reliance of marten on less profitable or high‐risk prey that could be impacting juvenile recruitment (Carlson et al., 2014). It is also possible that other mesocarnivores are limiting the distribution of marten by impacting juvenile survival or some other vital demographic rate (e.g., recruitment, growth; Louthan et al., 2015) that was not explicitly measured in the study, and this should be examined more fully in the future.

Local occupancy of marten within units was positively associated with the amount of canopy cover which may help limit their risk of predation (Eriksson et al., 2019). Detectability of marten was associated with higher percentages of deciduous forest in units and lower percentages of coniferous forest. The reasons for these associations are not entirely clear but it could be driven by differences in marten abundance in these two habitat types.

Interspecific competition and predation among co‐occurring carnivores can play an important role in determining community structure (Schoener, 1985). Other mesocarnivores are known to negatively impact marten (Storch et al., 1990; Thompson, 1994) but we did not detect significant species interaction factors in the models. Our findings are more similar to several recent studies that did not detect significant associations between marten and other mesocarnivores (Croose et al., 2019; Evans & Mortelliti, 2022; Manlick et al., 2017).

Fisher may be important intraguild predators of marten (Hodgman et al., 1994; Raine, 1981) and their impact has been suggested as a contributing factor to the apparent failed reintroduction of marten in Vermont (Moruzzi et al., 2003). Co‐occurrence patterns of marten and fisher (Zielinski et al., 2017) have been attributed to spatiotemporal segregation, differential habitat selection, and contrasting use of snow‐cover characteristics (Fisher et al., 2013; Krohn et al., 1997; Pauli et al., 2022), yet these were not well‐supported by the data. Fisher presence did not influence marten occupancy or detectability in the study area, and this supported our prediction. We observed a pattern of niche overlap between marten and fisher similar to other recent studies in the Great Lakes region and Maine that found substantial overlap in space and time, and neither species exhibited selection for separate habitats (Croose et al., 2019; Evans & Mortelliti, 2022; Manlick et al., 2017). Similar preferences during winter for a limited number of relatively abundant prey species could also explain the niche overlap seen in the present study between marten and fisher but to verify this, we would need to conduct additional dietary studies. Niche overlap is predicted to increase when resources are abundant (Schoener, 1982) and marten and fisher may simply synchronize nocturnal activity to their prey species (Carothers & Jaksić, 1984; Zielinski et al., 1983).

Marten may use several interacting mechanisms to reduce competition and predation. The pattern of marten detection times was multi‐modal which could indicate they are timing activity when other mesocarnivores are less active (Figure 4). We also found that marten uses vigilant antipredator behaviors (e.g., upright body position and short times at cameras; Sharpe & Van Horne, 1998) that others have attributed to a larger strategy for predator avoidance (Kautz et al., 2021; Zielinski et al., 2017). Marten, like many small‐bodied predators, may be forced to make a trade‐off between time spent foraging and time spent performing vigilant behaviors to limit the risk of predation (Wikenros et al., 2014). This may be particularly important for marten whose activity period largely overlapped with fisher.

## 6 MANAGEMENT IMPLICATIONS

The marten population in southern Vermont is of considerable conservation concern given their endangered status in the state and localized presence on the GMNF (O'Brien et al., 2018) where land management is carried out for multiple uses and not specifically for marten recovery. The limited distance that marten have expanded away from past release sites suggests at least two possible and potentially related explanations relevant to management; that marten may not be abundant in southern Vermont or that mesocarnivores are limiting the distribution of marten by impacting juvenile survival or some other vital demographic rate. Future efforts to promote marten recovery should consider the potential ameliorating effects of adjusting current harvesting levels of fisher and other mesocarnivores on interspecific competition and predation of marten.

Marten may occur at low abundance in southern Vermont, but this would need to be verified with additional studies. At present, there is not enough information to verify that recovery goals have been met for the southern Vermont marten population or to consider the establishment of a regulated annual harvest. A recent genetic assessment of the southern Vermont marten population found substantially lower genetic diversity there compared to other marten populations in the Northeast (Aylward et al., 2019). Low genetic diversity is associated with small population size. Based on our distribution data, marten appears to be regionally isolated from the two closest marten populations in New Hampshire (150 km) and New York (97 km). Small, isolated populations are more susceptible to demographic stochasticity and the genetic effects of inbreeding associated with drift. Consideration should be given to the feasibility of maintaining connective corridors between marten populations in northern Vermont and neighboring states.

Finally, we recommend promoting canopy cover through uneven‐aged forestry practices that have a positive association with marten presence including the maintenance of large tracts of undisturbed forest with structural complexity that promotes canopy cover. We view the parameter estimates from the models as a 3‐year snapshot over the sample season. We recommend implementing a long‐term monitoring program for marten using 3‐year trends in marten occupancy and detectability to verify their status in the future.

## AUTHOR CONTRIBUTIONS

**Paul Hapeman:** Conceptualization (lead); data curation (supporting); formal analysis (lead); funding acquisition (lead); investigation (supporting); methodology (lead); project administration (lead); supervision (lead); visualization (lead); writing – original draft (lead); writing – review and editing (equal). **Sarah Ashbrook:** Conceptualization (supporting); data curation (lead); formal analysis (supporting); funding acquisition (supporting); investigation (lead); methodology (supporting); project administration (supporting); supervision (supporting); visualization (supporting); writing – original draft (supporting); writing – review and editing (equal).

## CONFLICT OF INTEREST STATEMENT

The authors declare no conflicts of interest.

## Supporting information


Figure S1.

Table S1.

Table S2.

## Data Availability

Requests for access to the data should be made to one of the authors.

## References

[ece310904-bib-0001] Aragón, P. , & Sánchez‐Fernández, D. (2013). Can we disentangle predator–prey interactions from species distributions at a macro‐scale? A case study with a raptor species. Oikos, 122, 64–72.

[ece310904-bib-0002] Aylward, C. M. , Murdoch, J. D. , & Kilpatrick, C. W. (2019). Genetic legacies of translocation and relictual populations of American marten at the southeastern margin of their distribution. Conservation Genetics, 20, 275–286.

[ece310904-bib-0003] Brander, R. , & Books, D. (1973). Return of the fisher. Natural History, 82, 52–57.

[ece310904-bib-0004] Brown, J. H. (1984). On the relationship between abundance and distribution of species. The American Naturalist, 124, 255–279.

[ece310904-bib-0005] Brown, J. H. , Mehlman, D. W. , & Stevens, G. C. (1995). Spatial variation in abundance. Ecology, 76, 2028–2043.

[ece310904-bib-0006] Bull, E. L. , & Heater, T. W. (2001). Resting and denning sites of American martens in northeastern Oregon. Northwest Science, 74, 179–185.

[ece310904-bib-0007] Burnham, K. P. , & Anderson, D. R. (2002). Model selection and multimodel inference: A practical information‐theoretic approach (2nd ed.). Springer‐Verlag.

[ece310904-bib-0008] Buskirk, S. W. , & Powell, R. A. (1994). Habitat ecology of fishers and American martens. In S. W. Buskirk , A. Harestad , & M. Raphael (Eds.), Martens, sables, and fishers: Biology and conservation (pp. 283–296). Cornell University Press.

[ece310904-bib-0009] Carlson, J. E. , Gilbert, J. H. , Pokallus, J. W. , Manlick, P. J. , Moss, W. E. , & Pauli, J. N. (2014). Potential role of prey in the recovery of American martens to Wisconsin. Journal of Wildlife Management, 78, 1499–1504.

[ece310904-bib-0010] Carothers, J. H. , & Jaksić, F. M. (1984). Time as a niche difference: The role of interference competition. Oikos, 42, 403–406.

[ece310904-bib-0011] Chapin, T. G. , Harrison, D. J. , & Katnik, D. D. (1998). Influence of landscape pattern on habitat use by American marten in an industrial forest. Conservation Biology, 12, 1327–1337.

[ece310904-bib-0012] Cheveau, M. , Imbeau, L. , Drapeau, P. , & Belanger, L. (2013). Marten space use and habitat selection in managed coniferous boreal forests of eastern Canada. Journal of Wildlife Management, 77, 749–760.

[ece310904-bib-0013] Clark, T. , Anderson, E. , Douglas, C. , & Strickland, M. (1987). Martes americana. Mammalian Species, 289, 1–8.

[ece310904-bib-0016] Croose, E. , Bled, F. , Fowler, N. L. , Beyer, D. E., Jr. , & Belant, J. L. (2019). American marten and fisher do not segregate in space and time during winter in a mixed‐forest system. Ecology and Evolution, 9, 4906–4916. 31031953 10.1002/ece3.5097 PMC6476749

[ece310904-bib-0017] Dechner, A. , Flesher, K. , Lindell, C. , Veiga de Oliveira, T. , & Maurer, B. A. (2018). Determining carnivore habitat use in a rubber/forest landscape in Brazil using multispecies occupancy models. PLoS One, 13, e0197397. 29742171 10.1371/journal.pone.0197397 PMC5942838

[ece310904-bib-0018] Distefano, J. J. , Royar, K. J. , Pence, D. M. , & Denoncour, J. E. (1990). Marten recovery plan for Vermont. Vermont Fish & Wildlife Department and USFS‐GMNF.

[ece310904-bib-0019] Doherty, P. F. , White, G. C. , & Burnham, K. P. (2012). Comparison of model building and selection strategies. Journal of Ornithology, 152, S317–S323.

[ece310904-bib-0020] Donovan, T. , & Hines, J. (2007). Exercises in occupancy modeling and estimation. Electronic book.

[ece310904-bib-0021] Eriksson, C. E. , Moriarty, K. M. , Linnell, M. A. , & Levi, T. (2019). Biotic factors influencing the unexpected distribution of a Humboldt marten (*Martes caurina humboldtensis*) population in a young coastal forest. PLoS One, 14, e0214653. 31042737 10.1371/journal.pone.0214653 PMC6493723

[ece310904-bib-0022] Evans, B. E. , & Mortelliti, A. (2022). Effects of forest disturbance, snow depth, and intraguild dynamics on American marten and fisher occupancy in Maine, USA. Ecosphere, 13, e4027.

[ece310904-bib-0023] Fisher, J. T. , Bradbury, S. , B. , Anholt, L. R. , Volpe, J. , & Wheatley, M. (2013). Wolverines (*Gulo luscus*) on the Rocky Mountain slopes: Natural heterogeneity and landscape alteration as predictors of distribution. Canadian Journal of Zoology, 91, 706–716.

[ece310904-bib-0024] Foresman, K. R. , & Pearson, D. E. (1999). Activity patterns of American martens, *Martes americana*, snowshoe hares, *Lepus americanus*, and red squirrels, *Tamiasciurus hudsonicus*, in Westcentral Montana. Canadian Field‐Naturalist, 113, 386–389.

[ece310904-bib-0025] Glasser, J. W. (1982). A theory trophic strategies: The evolution of facultative specialist. The American Naturalist, 119, 250–262.

[ece310904-bib-0026] Godbout, G. , & Ouellet, J. P. (2010). Fine‐scale habitat selection of American marten at the southern fringe of the boreal forest. Ecoscience, 17, 175–185.

[ece310904-bib-0027] Gompper, M. E. , Lesmeister, D. B. , Ray, J. C. , Malcolm, J. R. , & Kays, R. (2016). Differential habitat use or intraguild interactions: What structures a carnivore community? PLoS One, 11, e0146055. 26731404 10.1371/journal.pone.0146055 PMC4711579

[ece310904-bib-0028] Gosse, J. W. , Cox, R. , & Avery, S. W. (2005). Home‐range characteristics and habitat use by American martens in eastern Newfoundland. Journal of Mammalogy, 86, 1156–1163.

[ece310904-bib-0029] Grassel, S. M. , Rachlow, J. L. , & Williams, C. J. (2015). Spatial interactions between sympatric carnivores: Asymmetric avoidance of an intraguild predator. Ecology and Evolution, 5, 2762–2773. 26306165 10.1002/ece3.1561 PMC4541984

[ece310904-bib-0030] Grassman, L. I., Jr. , Tewes, M. E. , Silvy, N. J. , & Kreeti‐yutanont, K. (2005). Spatial organization and diet of leopard cat (*Prionailurus bengalensis*) in northern‐central Thailand. Journal of Zoology, 266, 45–54.

[ece310904-bib-0031] Guo, K. , Liu, H. , Bao, H. , Hu, J. , Wang, S. , Zhang, W. , Zhao, Y. , & Jiang, Z. (2017). Habitat selection and their interspecific interactions for mammal assemblage in the greater Khingan Mountains, northeastern China. Wildlife Biology, 17, 1–8.

[ece310904-bib-0032] Hairston, N. G. , Nishikawa, K. C. , & Stenhouse, S. L. (1987). The evolution of competing species of terrestrial salamanders: Niche partitioning or interference? Evolutionary Ecology, 1, 247–262.

[ece310904-bib-0033] Hines, J. E. (2006). PRESENCE‐software to estimate patch occupancy and related parameters . USGS‐PWRC. http://www.mbr‐pwrc.usgs.gov/software/presence.html

[ece310904-bib-0034] Hodgman, T. P. , Harrison, D. J. , Katnik, D. D. , & Elowe, K. D. (1994). Survival in an intensively trapped marten population in Maine. Journal of Wildlife Management, 58, 593–600.

[ece310904-bib-0035] Jaeger, R. G. (1971a). Competitive exclusion as a factor influencing the distributions of two species of terrestrial salamanders. Ecology, 52, 632–637. 28973807 10.2307/1934151

[ece310904-bib-0036] Jaeger, R. G. (1971b). Moisture as a factor influencing the distributions of two species of terrestrial salamanders. Oecologia, 6, 191–207. 28310969 10.1007/BF00344914

[ece310904-bib-0037] Jaeger, R. G. (1972). Food as a limited resource in competition between two species of terrestrial salamanders. Ecology, 53, 535–546.

[ece310904-bib-0038] Jensen, P. G. (2012). Ecology of American martens in northern hardwood forests: Resource pulses and resource selection across temporal and spatial scales . Ph.D. Dissertation, McGill University, Montreal, Canada.

[ece310904-bib-0039] Johnson, C. A. , Fryxell, J. M. , Thompson, I. D. , & Baker, J. A. (2009). Mortality risk increases with natal dispersal distance in American martens. Proceedings of the Royal Society of London Biological Science B, 276, 3361–3367. 10.1098/rspb.2008.1958 PMC2817161 19570789

[ece310904-bib-0040] Kalle, R. , Ramesh, T. , Qureshi, Q. , & Sankar, K. (2013). Predicting the distribution pattern of small carnivores in response to environmental factors in the western Ghats. PLoS One, 8, e79295. 24244470 10.1371/journal.pone.0079295 PMC3828364

[ece310904-bib-0041] Kautz, T. M. , Beyer, D. E., Jr. , Farley, Z. , Kellner, N. L. K. F., III , Lutto, A. L. , Petroelje, T. R. , & Belant, J. L. (2021). American martens use vigilance and short‐term avoidance to navigate a landscape of fear from fishers at artificial scavenging sites. Scientific Reports, 11, 12146. 34108524 10.1038/s41598-021-91587-4 PMC8190286

[ece310904-bib-0042] Krohn, W. B. (2012). Distribution changes of American martens and fishers in eastern North America, 1699–2001. In K. B. Aubry , W. J. Zielinski , M. G. Raphael , G. Proulx , & S. W. Buskirk (Eds.), Biology and conservation of martens, sables, and fishers: A new synthesis (pp. 58–73). Cornell University Press.

[ece310904-bib-0043] Krohn, W. B. , Elowe, K. D. , & Boone, R. B. (1995). Relations among fishers, snow, and martens: Development and evaluation of two hypotheses. The Forestry Chronicle, 71, 97–105.

[ece310904-bib-0044] Krohn, W. B. , Zielinski, W. J. , & Boone, R. B. (1997). Relations among fishers, snow, and martens in California: Results from small‐scale spatial comparisons. In G. Proulx , H. N. Bryant , & P. M. Woodard (Eds.), Martes: Taxonomy, ecology, techniques and management. Proceedings of the Second International Martes Symposium (pp. 211–232). Provincial Museum of Alberta.

[ece310904-bib-0045] Kyaw, P. P. , Macdonald, D. W. , Penjor, U. , Htun, S. , Naing, H. , Burnham, D. , Kaszta, Z. , & Cushman, S. A. (2021). Investigating carnivore guild structure: Spatial and temporal relationships amongst threatened felids in Myanmar. International Journal of Geo‐Information, 10, 808.

[ece310904-bib-0046] Lambert, J. D. , Curran, Z. J. , & Reitsma, L. R. (2017). Guidelines for managing American marten habitat in New York and northern New England. High Branch Conservation Services.

[ece310904-bib-0048] Lesmeister, D. B. , Nielsen, C. K. , Schauber, E. M. , & Hellgren, E. C. (2015). Spatial and temporal structure of a mesocarnivore guild in midwestern North American. Wildlife Monographs, 191, 1–61.

[ece310904-bib-0049] Long, R. A. , Donovan, T. M. , MacKay, P. , Zielinski, W. J. , & Buzas, J. (2010). Predicting carnivore occurrence with noninvasive surveys and occupancy modeling. Landscape Ecology, 26, 327–340.

[ece310904-bib-0050] Louthan, A. M. , Doak, D. F. , & Angert, A. L. (2015). Where and when do species interactions set range limits? Trends in Ecology & Evolution, 12, 780–792. 10.1016/j.tree.2015.09.011 26525430

[ece310904-bib-0051] MacKenzie, D. L. , Nichols, J. D. , Royle, J. A. , Pollock, K. H. , Bailey, L. , & Hines, J. E. (2006). Occupancy estimation and modeling. Academic Press.

[ece310904-bib-0052] MacKenzie, D. L. , Nichols, J. D. , Sutton, N. , Kawanishi, K. , & Bailey, L. (2005). Improving inferences in population studies of rare species that are detected imperfectly. Ecology, 86, 1101–1113.

[ece310904-bib-0053] MacKenzie, D. L. , & Royle, J. D. (2005). Designing occupancy studies: General advice and allocating survey effort. Journal of Applied Ecology, 42, 1105–1114.

[ece310904-bib-0054] Manlick, P. , Woodford, J. E. , Zuckerberg, B. , & Pauli, J. N. (2017). Niche compression intensifies competition between reintroduced American martens (*Martes Americana*) and fishers (*Pekania pennanti*). Journal of Mammalogy, 98, 690–702.

[ece310904-bib-0055] Martin, E. M. , Delheimer, M. S. , Gabriel, M. W. , Wengert, G. M. , & Moriarty, K. M. (2022). Combined field and clinical methods clarify mortality causes and survival patterns of Pacific martens. Journal of Wildlife Management, 86, e22131. 10.1002/jwmg.22131

[ece310904-bib-0056] Meiri, S. , Dayan, T. , Simberloff, D. , & Grenyer, R. (2009). Life on the edge: Carnivore body size variation is all over the place. Proceedings of the Royal Society of London Biological Science B, 276, 1469–1476. 10.1098/rspb.2008.1318 PMC2677232 19324818

[ece310904-bib-0057] Mitchell, M. S. , & Hebblewhite, M. (2012). Carnivore habitat ecology: Integrating theory and application. In L. Boitani & R. A. Powell (Eds.), Carnivore ecology and conservation: A handbook of techniques (pp. 218–255). Oxford University Press.

[ece310904-bib-0058] Morin, D. J. , Yackulic, C. B. , Diffendorfer, J. E. , Lesmeister, D. B. , Nielsen, C. K. , Reid, J. E. , & Schauber, E. M. (2020). Is your ad hoc model selection strategy affecting your multimodel inference? Ecosphere, 11, e02997.

[ece310904-bib-0059] Moruzzi, T. L. , Royar, K. J. , Gove, C. , Brooks, R. T. , Bernier, C. , Thompson, F. L. , DeGraaf, R. M. , & Fuller, T. K. (2003). Assessing an American marten, *Martes americana*, reintroduction in Vermont. Canadian Field‐Naturalist, 117, 190–195.

[ece310904-bib-0060] Mott, C. L. (2010). Environmental constraints to the geographic expansion of plant and animal species. Nature Education Knowledge, 3, 72.

[ece310904-bib-0061] O'Brien, P. J. , Bernier, C. , & Hapeman, P. (2018). A new record of an American marten (*Martes americana*) population in southern Vermont. Small Carnivore Conservation, 56, 68–75.

[ece310904-bib-0062] O'Connor, K. M. , Nathan, L. R. , Liberati, M. R. , Tingley, M. W. , Vokoun, J. C. , & Rittenhouse, T. A. G. (2017). Camera trap arrays improve detection probability of wildlife: Investigating study design considerations using an empirical dataset. PLoS One, 12, e0175684. 28422973 10.1371/journal.pone.0175684 PMC5396891

[ece310904-bib-0064] Pauli, J. N. , Manlick, P. J. , Tucker, J. M. , G. B. , Smith, P. G. J. , & Fisher, J. T. (2022). Competitive overlap between martens *Martes americana* and *Martes caurina* and fishers *Pekania pennanti*: A rangewide perspective and synthesis. Mammal Review, 52, 392–409.

[ece310904-bib-0065] Pavlacky, D. C., Jr. , Blakesley, J. A. , White, G. C. , Hanni, D. J. , & Lukacs, P. M. (2012). Hierarchical multi‐scale occupancy estimation for monitoring wildlife populations. Journal of Wildlife Management, 76, 154–162.

[ece310904-bib-0066] Payer, D. C. , & Harrison, D. J. (2004). Relationships between forest structure and habitat use by American martens in Maine, USA. In D. J. Harrison , A. K. Fuller , & G. Proulx (Eds.), Martens and fishers (Martes) in human‐altered environments (pp. 173–186). Springer‐Verlag.

[ece310904-bib-0067] Powell, R. A. , Buskirk, S. W. , & Zielinski, W. J. (2003). Fisher (*Martes pennanti*) and marten (*Martes americana*). In G. Feldhamer , B. C. Thompson , & J. A. Chapman (Eds.), Wild mammals of North America: Biology, management, and conservation (2nd ed., pp. 635–649). Johns Hopkins University Press.

[ece310904-bib-0068] Raine, R. M. (1981). Winter food habits, responses to snow cover and movements of fisher (Martes pennanti) and marten (Martes americana) in south‐eastern Manitoba . MS Thesis, University of Manitoba, Winnepeg, Canada.

[ece310904-bib-0069] Rhoads, S. (1903). The mammals of Pennsylvania and New Jersey ‐ a biographic, historic and descriptive account of the furred animals of land and sea, both living and extinct, known to have existed in these states . Publisher unknown, Philadelphia, Pennsylvania.

[ece310904-bib-0070] Richmond, O. , Hines, J. E. , & Beissinger, S. R. (2010). Two‐species occupancy models: A new parameterization applied to co‐occurrence of secretive rails. Ecological Applications, 20, 2036–2046. 21049888 10.1890/09-0470.1

[ece310904-bib-0071] Ridout, M. S. , & Linkie, M. (2009). Estimating overlap of daily activity patterns from camera trap data. Journal of Agricultural, Biological, and Environmental Statistics, 14, 322–337.

[ece310904-bib-0072] Royar, K. J. (1992). Monitoring reintroduced marten populations in Vermont . Vermont Fish and Wildlife Department.

[ece310904-bib-0073] Schoener, T. W. (1982). The controversy over interspecific competition. American Scientist, 70, 27–39.

[ece310904-bib-0074] Schoener, T. W. (1985). Some comments on Connell's and my reviews of field experiments on interspecific competition. The American Naturalist, 125, 730–740.

[ece310904-bib-0075] Sharpe, P. B. , & Van Horne, B. (1998). Influence of habitat on the behaviour of Townsend's ground squirrels (*Spermophilus townsendii*). Journal of Mammalogy, 79, 906–918.

[ece310904-bib-0076] Siccama, T. (2014). Vegetation, soil, and climate on the Green Mountains of Vermont. Ecological Monographs, 44, 325–349.

[ece310904-bib-0077] Silver, H. (1957). A history of New Hampshire game and furbearers. New Hampshire Fish and Game Department.

[ece310904-bib-0078] Sirén, A. P. K. , & Morelli, T. L. (2020). Interactive range‐limit theory (iRLT): An extension for predicting range shifts. Journal of Animal Ecology, 89, 940–954. 31758805 10.1111/1365-2656.13150 PMC7187220

[ece310904-bib-0079] Sirén, A. P. K. , Perkins, P. J. , Ducey, M. J. , & Kilborn, J. R. (2016). Spatial ecology and resource selection of a high‐elevation American marten (*Martes americana*) population in the northeastern United States. Canadian Journal of Zoology, 94, 169–180.

[ece310904-bib-0080] Smith, A. C. , & Schaefer, J. A. (2002). Home‐range size and habitat selection by American marten (*Martes americana*) in Labrador. Canadian Journal of Zoology, 80, 1602–1609.

[ece310904-bib-0081] Smith, J. A. , Thomas, A. A. , Levi, T. , Wang, Y. , & Wilmers, C. C. (2018). Human activity reduces niche partitioning among three widespread mesocarnivores. Oikos, 127, 890–901.

[ece310904-bib-0082] St. Pierre, C. , Ouellet, J. P. , & Crête, M. (2006). Do competitive intraguild interactions affect space and habitat use by small carnivores in a forested landscape? Ecography, 29, 487–496.

[ece310904-bib-0083] Storch, I. , Lindström, E. , & de Jounge, J. (1990). Habitat selection and food habits of the pine marten in relation to competition with the red fox. Acta Theriologica, 35, 311–320.

[ece310904-bib-0084] Suffice, P. , Mazerolle, M. J. , Imbeau, L. , Cheveau, M. , Asselin, H. , & Drapeau, P. (2023). Site occupancy by American martens and fishers in temperate deciduous forests of Québec. Journal of Mammalogy, 104, 159–170. 36818684 10.1093/jmammal/gyac092 PMC9936503

[ece310904-bib-0085] Thompson, I. D. (1994). Marten population in uncut and logged boreal forests in Ontario. Journal of Wildlife Management, 58, 272–280.

[ece310904-bib-0086] Uboni, A. , Smith, D. W. , Stahler, D. R. , & Vucetich, J. A. (2017). Selecting habitat to what purpose? The advantage of exploring the habitat–fitness relationship. Ecosphere, 8, e01705.

[ece310904-bib-0087] United States Department of Agriculture Forest Service (USFS) . (2006). Green Mountain National Forest Land and Resource Management Plan . pp. 1–158. https://www.google.com/url?sa=t&rct=j&q=&esrc=s&source=web&cd=&ved=2ahUKEwjhuYSljIOEAxUknokEHTzsAggQFnoECBkQAQ&url=https%3A%2F%2Fwww.fs.usda.gov%2FInternet%2FFSE_DOCUMENTS%2Fstelprdb5334042.pdf&usg=AOvVaw083HRevA0vCQ4yQ2bAmjes&opi=89978449

[ece310904-bib-0088] United States Department of Agriculture Forest Service (USFS) . (2015). Region 5 Common Stand Exam Field Guide . pp. 1–114. https://www.fs.usda.gov/nrm/fsveg/index.shtml

[ece310904-bib-0089] Vanek, A. T. , Fortin, D. , Thaker, M. , Ogden, M. , Owen, C. , Greatwood, S. , & Slotow, R. (2013). Moving to stay in place: Behavioral mechanisms for coexistence of African large carnivores. Ecology, 94, 2619–2631. 24400513 10.1890/13-0217.1

[ece310904-bib-0090] Vernam, D. J. (1987). Marten habitat use in the Bear Creek burn, Alaska . M.S. Thesis, University of Alaska‐Fairbanks, Fairbanks, Alaska.

[ece310904-bib-0091] White, G. C. , & Burnham, K. P. (1999). Program MARK: Survival estimation from populations of marked animals. Bird Study, 46, 120–138.

[ece310904-bib-0092] Whittington, J. , Hebblewhite, M. , Baron, R. W. , Ford, A. T. , & Paczkow, J. (2022). Towns and trails drive carnivore movement behaviour, resource selection, and connectivity. Movement Ecology, 10, 17. 35395833 10.1186/s40462-022-00318-5 PMC8994267

[ece310904-bib-0093] Wikenros, C. , Stahlberg, S. , & Sand, H. (2014). Feeding under high risk of intraguild predation: Vigilance patterns of two medium‐sized generalist predators. Journal of Mammalogy, 95, 862–870.

[ece310904-bib-0094] Wilson, T. L. , & Schmidt, J. H. (2015). Scale dependence in occupancy models: Implications for estimating bear den distribution and abundance. Ecosphere, 6, 1–13.

[ece310904-bib-0095] Zielinski, W. J. , Spencer, W. D. , & Barrett, R. H. (1983). Relationship between food habits and activity patterns of pine martens. Journal of Mammalogy, 64, 387–396.

[ece310904-bib-0096] Zielinski, W. J. , Tucker, J. M. , & Rennie, K. M. (2017). Niche overlap of competing carnivores across climatic gradients and the conservation implications of climate change at geographic range margins. Biological Conservation, 209, 533–545.

