# Supplementary figures and images for: American marten occupancy and activity patterns at the southern extent of their range in the eastern United States
Source: Ecol Evol. 2024 Feb 5;14(2):e10904. doi: 10.1002/ece3.10904 (PMC10844684; doi:10.1002/ece3.10904)

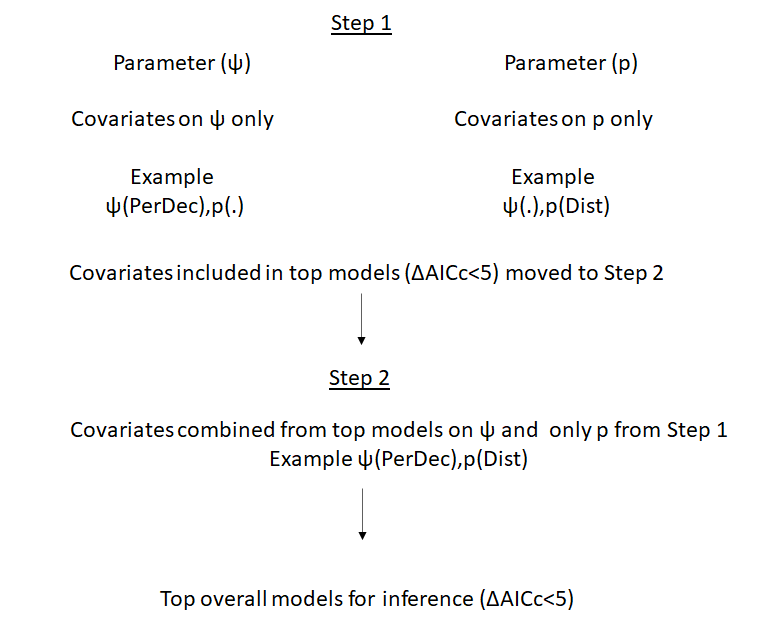

Supplement: Supplementary file 1 — Figure S1. [file ECE3-14-e10904-s003.tif]
