# Supplementary material for: American marten occupancy and activity patterns at the southern extent of their range in the eastern United States
Source: Ecol Evol. 2024 Feb 5;14(2):e10904. doi: 10.1002/ece3.10904 (PMC10844684; doi:10.1002/ece3.10904)
Supplement: Supplementary file 2 — Table S1. [file ECE3-14-e10904-s001.docx]

Table S1: Beta estimates, standard errors, and bounds of the 95% confidence intervals for the 5 top until level occupancy models and single top multiscale model.

| **Model** | **Beta Estimate** | **Standard Error** | **Lower CI** | **Upper CI** |
| --- | --- | --- | --- | --- |
| Psi(.),p(Dist+PerDec) |  |  |  |  |
| p (Intercept) | 0.38 | 0.358 | -0.321 | 1.082 |
| p (Dist) | 2.278 | 0.591 | -3.436 | -1.121 |
| P (PerDec) | 1.158 | 0.439 | 0.298 | 2.017 |
| Psi (Intercept) | 0.413 | 0.409 | -0.388 | 1.214 |
|  |  |  |  |  |
| Psi(Dist),p(PerCC) |  |  |  |  |
| p (Intercept) | 0.714 | 0.363 | 0.002 | 1.426 |
| p (PerCC) | 1.243 | 0.41 | 0.439 | 2.048 |
| Psi (Intercept) | 0.088 | 0.433 | -0.761 | 0.938 |
| Psi (Dist) | -1.225 | 0.493 | -2.193 | -0.257 |
|  |  |  |  |  |
| Psi(.),p(Dist+PerCC) |  |  |  |  |
| p (Intercept) | -0.016 | 0.368 | -0.738 | 0.705 |
| p (Dist) | -1.576 | 0.442 | -2.444 | -0.709 |
| p (PerCC) | 1.129 | 0.403 | 0.339 | 1.920 |
| Psi (Intercept) | 0.578 | 0.463 | -0.330 | 1.487 |
|  |  |  |  |  |
| Psi(.),p(Dist+PerCon) |  |  |  |  |
| p (Intercept) | 0.384 | 0.35 | -0.303 | 1.071 |
| p (Dist) | -1.877 | 0.482 | -2.823 | -0.931 |
| p (PerCon) | -0.802 | 0.322 | -1.433 | -0.17 |
| Psi (Intercept) | 0.403 | 0.404 | -0.39 | 1.197 |
|  |  |  |  |  |
| Psi(Dist),p(PerDec) |  |  |  |  |
| p (Intercept) | 1.261 | 0.408 | 0.461 | 2.061 |
| p (PerDec) | 0.955 | 0.415 | 0.14 | 1.769 |
| Psi (Intercept) | -0.144 | 0.379 | -0.888 | 0.599 |
| Psi Dist) | -1.057 | 0.435 | -1.911 | -0.202 |
|  |  |  |  |  |
| Psi(.),Theta(PerCC),p(Dist) |  |  |  |  |
| Psi (Intercept) | 0.28 | 0.409 | -0.521 | 1.082 |
| Theta (Intercept) | 0.008 | 0.263 | -0.508 | 0.526 |
| Theta (PerCC) | 0.743 | 0.352 | 0.052 | 1.435 |
| p (Intercept) | -0.492 | 0.237 | -0.958 | -0.026 |
| p (Dist) | -1.452 | 0.297 | -2.035 | -0.87 |
