# Supplementary material for: American marten occupancy and activity patterns at the southern extent of their range in the eastern United States
Source: Ecol Evol. 2024 Feb 5;14(2):e10904. doi: 10.1002/ece3.10904 (PMC10844684; doi:10.1002/ece3.10904)
Supplement: Supplementary file 3 — Table S2. [file ECE3-14-e10904-s002.docx]

Table S2: Range of covariate values used in the unit level analysis.

| Covariate | Unit | Lower Bound | Upper Bound |
| --- | --- | --- | --- |
| PerFor | Percent | 86.31 | 100 |
| PerDec | Percent | 9.033 | 87.27 |
| PerMix | Percent | 2.33 | 59.85 |
| PerCon | Percent | 0.2 | 19.72 |
| PerWW | Percent | 0 | 24.81 |
| PerWater | Percent | 0 | 4.79 |
| PerHumDist | Percent | 0 | 7.02 |
| PerCC | Percent | 71.76 | 86.19 |
| Elevation | Meters | 545.57 | 903.42 |
| TRI | N/A | 0.468 | 0.502 |
| Snow | Centimeters | 4.46 | 69.44 |
| Dist | Kilometers | 0.64 | 25.35 |
